# Notch Controls Cell Adhesion in the Drosophila Eye

**DOI:** 10.1371/journal.pgen.1004087

**Published:** 2014-01-09

**Authors:** Sujin Bao

**Affiliations:** Saint James School of Medicine, Bonaire, Netherlands Antilles; University of North Carolina at Chapel Hill, United States of America

## Abstract

Sporadic evidence suggests Notch is involved in cell adhesion. However, the underlying mechanism is unknown. Here I have investigated an epithelial remodeling process in the *Drosophila* eye in which two primary pigment cells (PPCs) with a characteristic ‘kidney’ shape enwrap and eventually isolate a group of cone cells from inter-ommatidial cells (IOCs). This paper shows that in the developing *Drosophila* eye the ligand Delta was transcribed in cone cells and *Notch* was activated in the adjacent PPC precursors. In the absence of Notch, emerging PPCs failed to enwrap cone cells, and *hibris* (*hbs*) and *sns*, two genes coding for adhesion molecules of the Nephrin group that mediate preferential adhesion, were not transcribed in PPC precursors. Conversely, activation of Notch in single IOCs led to ectopic expression of *hbs* and *sns*. By contrast, in a single IOC that normally transcribes *rst*, a gene coding for an adhesion molecule of the Neph1 group that binds Hbs and Sns, activation of Notch led to a loss of *rst* transcription. In addition, in a Notch mutant where two emerging PPCs failed to enwrap cone cells, expression of *hbs* in PPC precursors restored the ability of these cells to surround cone cells. Further, expression of *hbs* or *rst* in a single *rst*- or *hbs*-expressing cell, respectively, led to removal of the counterpart from the membrane within the same cell through *cis*-interaction and forced expression of Rst in all *hbs*-expressing PPCs strongly disrupted the remodeling process. Finally, a loss of both *hbs* and *sns* in single PPC precursors led to constriction of the apical surface that compromised the ‘kidney’ shape of PPCs. Taken together, these results indicate that cone cells utilize *Notch* signaling to instruct neighboring PPC precursors to surround them and Notch controls the remodeling process by differentially regulating four adhesion genes.

## Author Summary

In developing tissues, one way to isolate a group of cells from the rest of the tissue is to induce a few neighboring cells to surround them. How centrally localized cells communicate with neighboring cells and how neighboring cells respond to signaling is not well understood. This work describes a mechanism underlying an epithelial remodeling process in the *Drosophila* eye in which two primary pigment cells (PPCs) with a characteristic ‘kidney’ shape enwrap and isolate a group of cone cells from inter-ommatidial cells (IOCs). This paper shows that cone cells utilize Notch signaling to communicate with neighboring PPC precursors. In response to Notch signaling, PPC precursors activate transcription of *hbs* and *sns*, two genes coding for adhesion molecules of the Nephrin group that bind Rst and Kirre, adhesion molecules of the Neph1 group. At the same time, PPC precursors inactivate transcription of *rst* and *kirre* genes. In addition, binding of Hbs or Rst to its counterpart from the same cell (*cis*-interaction) destabilizes the protein complex and promotes removal of the counterparts from the membrane, leading to complementary distribution of four adhesion molecules within two populations of cells. Thus, Notch controls epithelial remodeling by differentially regulating four adhesion genes.

## Introduction

Pattern formation in developing tissues requires cell signaling. A small number of signaling pathways are repeatedly utilized for cell fate decisions in developing tissues (reviewed in [1]). In addition, cell signaling is also known to play a role in controlling cell sorting. For example, in the *Drosophila* wing, Hh signaling regulates cell segregation between anterior and posterior compartments (reviewed in [2]), while Notch signaling is required for establishing a boundary that separates dorsal and ventral cells (reviewed in [3]). In the *Drosophila* eye, Notch is required for a variety of developmental steps including rearranging pigment cells into hexagonal arrays [4]. All these observations raise the question of how Notch is involved in tissue remodeling. The observation that Notch is expressed in an epithelial sheet in the *Drosophila* embryo and continuously required for embryonic development after cell fate decision has led to speculation that Notch is involved in cell adhesion [5]. The behavior of primary pigment cells in the pupal eye also supports this view [4]. However, how Notch is involved in cell adhesion remains unclear.

Evidence accumulated to date supports the notion that cell adhesion plays a direct role in tissue remodeling. As first noted by J. Holtfreter and later formulated in “Differential Adhesion Hypothesis” (DAH) by M. Steinberg: sorting behaviors of cells are driven by interfacial free energy arising from differential adhesion among cells [6], [7], [8], [9]. In vivo observations support the DAH model. For example, in the *Drosophila* egg chamber, differential expression of E-cadherin determines localization of oocytes [10], [11]. In the eye epithelium, homophilic interactions mediated by E- and N-cadherin direct a group of four cone cells to arrange in a pattern that minimizes surface free energy [12]. In the chick spinal cord, MN-cadherin is involved in sorting out motor neurons [13]. All these examples show that cadherins are directly responsible for cell sorting in a variety of tissues through homophilic interactions. On the other hand, more complex patterns involve more intricate mechanisms. For example, in the *Drosophila* pupal eye organizing pigment cells into hexagonal arrays requires two groups of heterophilic-interacting adhesion molecules: Hibris (Hbs) and Sticks-and-Stones (Sns) from the Nephrin group; Roughest (Rst) and Kin of Irre (Kirre) from the Neph1 group [14]. Nephrin and Neph1 are adhesion molecules of the IRM family within the immunoglobulin (Ig) superfamily and both proteins are essential for maintaining specialized junctions during kidney development in mammals [15]. Despite mounting evidence linking cell adhesion to cellular patterns, how cell-cell adhesion is regulated in developing tissues to generate a variety of cellular patterns remains unclear.

This work describes a mechanism underlying an epithelial remodeling process in the *Drosophila* eye in which two primary pigment cells (PPCs) enwrap and isolate a group of cone cells from inter-ommatidial cells (IOCs). This paper shows that *Notch* signaling controls transcription of two groups of adhesion genes in the *Drosophila* eye. Notch activates adhesion genes of the Nephrin group but suppresses those of the Neph1 group. Differential distribution of two groups of adhesion molecules is further facilitated by removal of one group of adhesion molecules by another group through *cis*-interactions, leading to complementary distribution of four adhesion molecules within two populations of cells. This work uncovers a link between cell signaling and tissue remodeling.

## Results

### 1. Notch is required for organization of ommatidial cells

**Figure 1:**
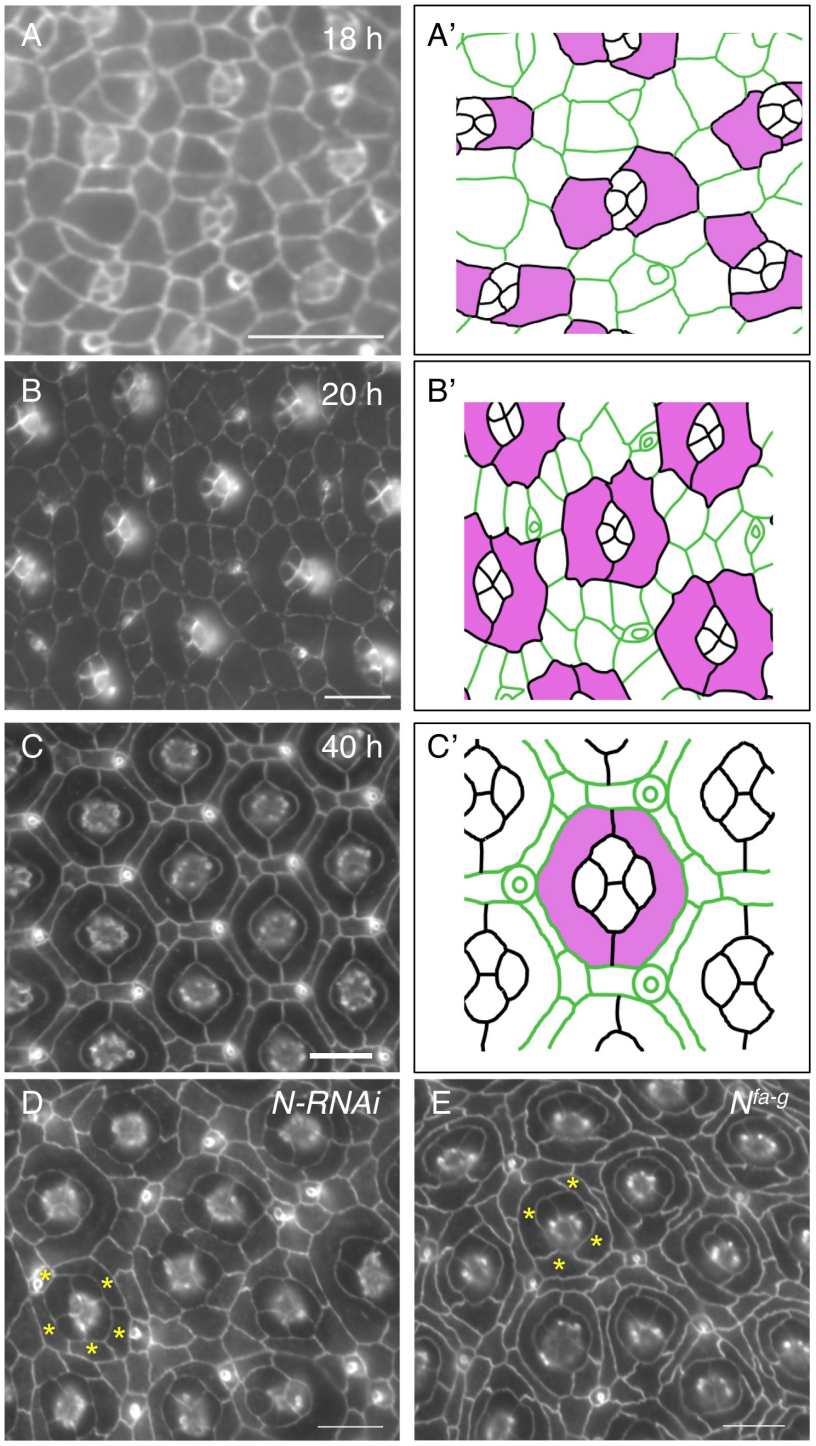
Notch is required for development of primary pigment cells (PPCs). Eyes were stained using an anti-E-cadherin antibody in this figure. A–C) Wild type eyes at 18 h (A), 20 h (B) and 40 h (C) are shown. The tracings of eyes are shown in A′–C′, where PPC or PPCs precursors are highlighted in pink. D) Knockdown of Notch using a Notch RNAi transgene led to a failure of PPCs to develop. Typically, 3–5 cells (asterisks) were found adjacent to a cone cell cluster at 40 h APF. E) In the *N^fa-g^* mutant, PPCs failed to develop. Frequently, 3–5 cells (asterisks) were contacting cone cells at 40 h APF. Scale bars, 10 µm.

The *Drosophila* eye derives from an invaginated epithelium at the embryonic stage [16]. Photoreceptor neurons and lens-secreting cone cells are specified at late larval and early pupal stages. At 18 h after puparium formation (APF), cone cells are surrounded by 4–5 inter-ommatidial cells (IOCs), which have relaxed apical profiles (Fig. 1A–A′). Shortly, two cells adjacent to cone cells start to expand apical contacts with cone cells in most ommatidia. At 20 h APF, these two cells completely enwrap cone cells with a ‘kidney’ shape and they become two primary pigment cells (PPCs) (Fig. 1B–B′). As a result, cone cells are fully isolated from the rest of IOCs within the epithelial plane. Further rearrangement of IOCs gives rise to a one-cell wide hexagonal lattice of IOCs that fully separates ommatidia. Separation of ommatidia by IOCs will eventually serve to optically insulate the ommatidial array across the eye (Fig. 1C–C′).

When Notch was depleted in all IOCs using RNAi, PPC precursors failed to enwrap cone cells. As a result, at 40 h APF, the cone cell cluster was found typically in direct contact with 4∼5 IOCs in an ommatidium (Fig. 1D), indicating Notch is required for the assembly of ommatidia (cone cells and PPCs). This phenotype is very reminiscent of the one seen in *N^fa-g^* (Fig. 1E). *N^fa-g^* is a loss-of-function *Notch* allele in which the activity of Notch is retained throughout larval stages but lost within the pupal stage [17]. As a result, cone cells in the eye are not affected by the mutation. Previous studies indicate that in *N^fa-g^* mutants two PPC precursors initially touch each other at both ends but they fail to establish contacts [4], [18], suggesting weakened adhesion between PPCs and/or adhesion between PPCs and cone cells. However, it has remained unclear how Notch is involved in cell-cell adhesion.

### 2. Notch is activated in PPCs

**Figure 2:**
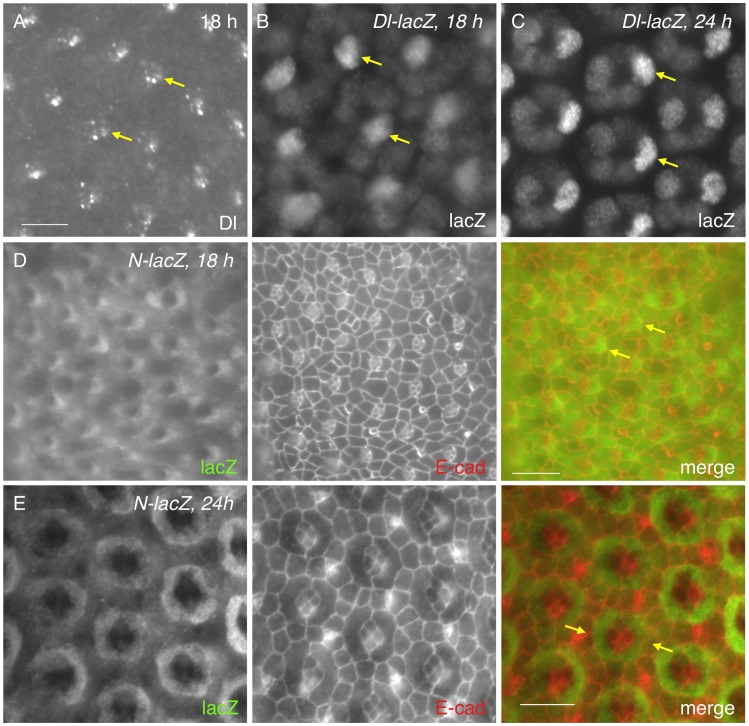
Notch is activated in PPC precursors. A) The Delta (Dl) protein was detected in cone cells (arrows) at 18 h APF as assessed with an anti-Dl antibody. B–C) Expression of a *Dl* reporter at 18 h (B) and 24 h APF (C) confirms its cone cell specificity. LacZ was detected at the highest level in anterior cone cells (arrows). D–E) Expression of *GBE-Su(H)_m8_-lacZ*, a reporter for Notch activity at 18 h (D) and 24 h APF (E). LacZ staining is shown on the left panel and E-cad channel in the middle. Merged views are shown on the right. Notch activity was high in cells adjacent to the anterior cone cells at 18 h APF (arrows, D). The difference of Notch activity between PPC precursors became less obvious at 24 h APF than earlier stages (arrows, E). Scale bars, 10 µm.

The receptor Notch is broadly expressed in all cells in the early pupal eye [19], [20], [21]. In contrast, expression of the ligand Delta (Dl) is often cell type- specific and the protein is predominantly found within endocytic vesicles [21]. Consistent with the previous study [21], Dl was detected in cone cells at 18 h APF (Fig. 2A). Using a *Dl*-specific reporter, *Dl* transcript was detected in cone cells (Fig. 2B). Especially, anterior cone cells had the highest level of *Dl* expression at this early stage (Fig. 2B). By 24 h APF, although expression in the posterior cone cells was slightly increased, *Dl* expression in the anterior cone cells still remained the highest within the cone cell cluster (Fig. 2C). To identify the cell types that receive active Notch signaling, a Notch activity reporter *GBE-Su(H)_m8_-lacZ* [22] was used. Consistent with expression of the *Dl* reporter, the Notch activity was detected in a significantly higher level in two cells adjacent to anterior-posterior cone cells than in other cells at 18 h APF (Fig. 2D). These two cells were presumably the two PPC precursors. In particular, the highest Notch reporter activity was detected in the PPC precursor adjacent to the anterior cone cell within each ommatidium (Fig. 2D). By 24 h APF, *GBE-Su(H)m8-lacZ* expression was found in both PPCs and the difference in the level of lacZ expression between these cells became less obvious than earlier stages (Fig. 2E). Therefore, *Dl* transcription within the anterior and posterior cone cells is correlated with a high level of the Notch activity in the two PPC precursors.

### 3. Notch signaling activates transcription of *hbs* and *sns*

**Figure 3:**
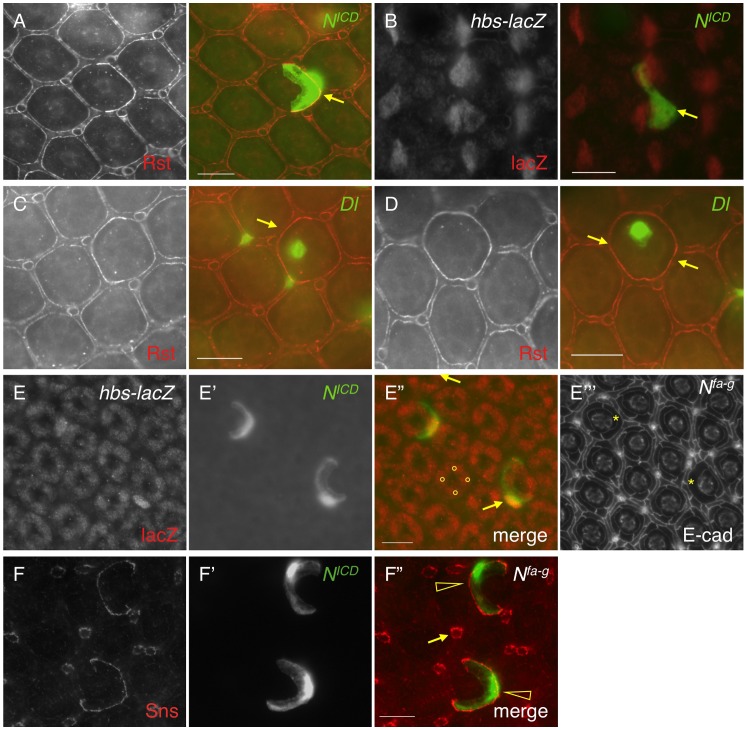
Notch signaling activates *hbs* and *sns* expression. A–D) *N^ICD^* or *Dl* (green) was over-expressed in single cells marked by GFP. The eyes were stained with an anti-Rst (left, A, C and D) or anti-lacZ antibody (left, B). Merged views are shown on the right. A) Over-expression of *N^ICD^* increased Rst on the membrane. Arrows point to a PPC-IOC border with elevated Rst. B) Ectopic *N^ICD^* induced ectopic *hbs* transcription as assessed using a *hbs* reporter (*hbs-lacZ*). Upon expression of *N^ICD^* in a single IOC, ectopic lacZ was observed (arrow). C) When *Dl* was over-expressed in a posterior cone cell, ectopic Rst was detected at the border between the posterior PPC and its neighboring IOCs (arrow). D) When *Dl* was over-expressed in a polar cone cell, ectopic Rst was found at all borders surrounding the two PPCs (arrows). E–E″′) Notch is required for *hbs* transcription. In the *N^fa-g^* mutant, *hbs* transcription (E) as assessed by the *hbs* reporter activity, was only detected in cone cells (bullets, E″) but lost in PPCs. In the *Notch* mutant, when *Notch* was activated in single IOCs by expressing *N^ICD^* (E′), *hbs* transcription (arrows) as well as the characteristic ‘kidney’ shape of PPCs (asterisks) was restored. The lacZ and *N^ICD^* channels are shown in E and E′, respectively, and the merged view in E″. The E-cadherin channel is shown in E″′. F–F″) Notch is required for *sns* expression. In the *N^fa-g^* mutant, the Sns protein (F) was lost in PPCs. Sns expression in bristle groups (arrows) was not affected. In this mutant, when Notch was activated in single IOCs by expressing *N^ICD^* (F′), the Sns protein (open arrowheads) was restored. The Sns channel is shown in F and the *N^ICD^* channel in F′. The merged view is shown in F″. Scale bars, 10 µm.

Previously it has been shown that *hbs* and *sns*, two genes from the Nephrin group, are transcribed in PPCs [14], [23]. The pattern of the Notch activity is very reminiscent of *hbs* and *sns* expression. When an intracellular domain of Notch (N^ICD^, an activated form of Notch) was expressed in a single PPC in the eye using a FLP-out technique [24], the Rst protein level was increased 128% at the border between the target PPC and neighboring IOCs compared with wild type borders (Fig. 3A and Table 1), a phenotype very similar to over-expression of *hbs* in a PPC [14]. To test whether *hbs* transcription was activated upon activation of Notch, *N^ICD^* was expressed in a single IOC. Upon activation of Notch, an ectopic activity of the *hbs* reporter P[w+]36.1 was observed in the target IOC (Fig. 3B). These results indicate Notch is sufficient to activate *hbs* transcription.

**Table 1:** Quantification of changes in the level of Rst or Hbs upon genetic manipulations.

Protein	Genetic manipulation	Intensity*		Note
		PPC-IOC	Change	
1. Rst	*wild type*	0.551±0.054	0.0	Fig. 3A
	*N^ICD^* in PPCs	1.258	+128%	
2. Rst	*wild type*	0.314±0.059	0.0	Fig. 3C
	*Dl* in cone cells	0.536	+70.9%	
3. Rst	*wild type*	0.437±0.092	0.0	Fig. 3D
	*Dl* in cone cells	0.791	+81.1%	
4. Rst	*wild type*	0.490±0.075	0.0	Fig. 4A
	*N^ICD^* in IOCs	0.294	−40.0%	
5. Kirre	*wild type*	0.595±0.082	0.0	Fig. 4D
	*N^ICD^* in IOCs	0.260	−56.2%	
6. Rst	*wild type*	0.379±0.056	0.0	Fig. 6A
	*hbs* in IOCs	0.141	−62.7%	
7. Hbs	*wild type*	1.414±0.227	0.0	Fig. 6B
	*rst* in PPCs	0.101	−92.8%	

Integrated density (ID) per unit length was used to calculate Intensity (I) of a protein at the PPC-IOC border. I = ID/L where L is the length of a given border in pixel. ID was measured using ImageJ as described in the Materials and Methods. Standard deviations are provided for control borders.

Consistently, when the Notch ligand Delta (Dl) was over-expressed in a single cone cell (either anterior or posterior), the Rst level was increased about 71% at the border between the adjacent PPC and its neighboring IOCs (Fig. 3C and Table 1). When Dl was over-expressed in a single polar or equatorial cone cell, the Rst level was elevated about 81% at the two PPC-IOC borders encircling two PPCs (Fig. 3D). These results indicate that the ligand Dl in cone cells is sufficient to activate *hbs* transcription in neighboring PPCs.

To test the necessity of Notch in control of *hbs* transcription, *N^fa-g^* mutant was used along with the *hbs* reporter *P[w+]36.1*. In the wild type eye, the *hbs* reporter was detected in emerging PPCs as well as in cone cells [14]. In *N^fa-g^* mutants, the *hbs* reporter activity was retained in cone cells but lost in PPC precursors at 40 h APF (Fig. 3E). When GFP alone was expressed in single IOCs in the *N^fa-g^* mutant, 12% of clones (n = 86, 4 eyes) exhibited a kidney-shape seen in wild type PPCs. In contrast, when *N^ICD^* (activated Notch) was expressed in single IOCs in the same mutant, 100% of clones (n = 92, 4 eyes) exhibited kidney shape (Fig. 3E″′). In addition, these cells also expressed the *hbs* reporter (Fig. 3E–E″). These results indicate that Notch signaling is required for activation of *hbs* transcription. A similar effect was also observed with *sns* when Notch was activated in single IOCs in the *N^fa-g^* mutant (Fig. 3F–F″). Taken together, these data indicate that Notch is both sufficient and necessary to activate transcription of both *hbs* and *sns*, the adhesion genes of the Nephrin group.

### 4. Notch signaling suppresses transcription of *rst* and *kirre*

**Figure 4:**
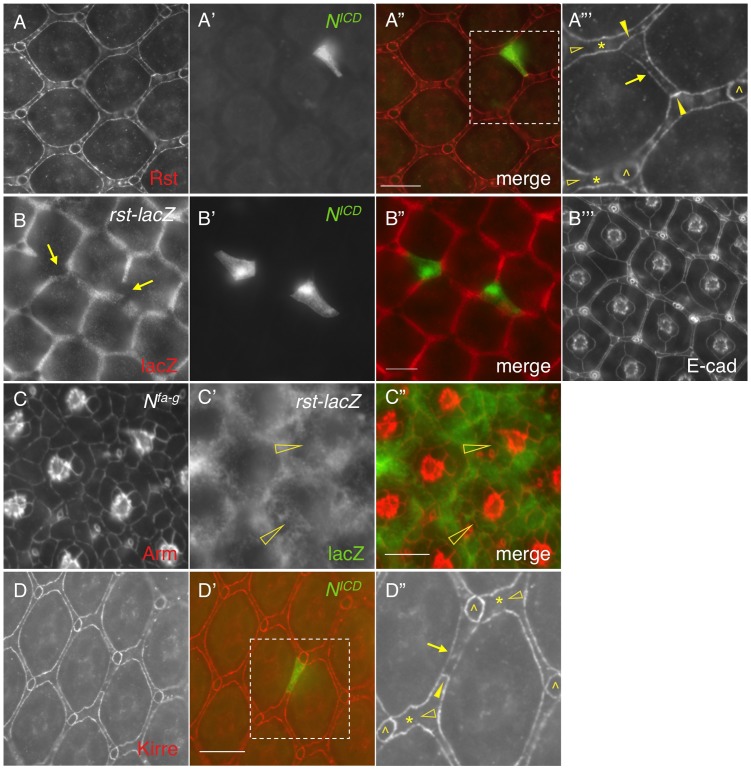
Notch signaling suppresses *rst* and *kirre* expression. A–A″′) *N^ICD^* (A′) was over-expressed in a single IOC and the Rst protein (A) was assessed using an anti-Rst antibody. The merged view is shown in A″. The enlarged view of a boxed region in A″ is shown in A″′. For clarity, only Rst channel is shown in A″′. Rst was increased at IOC-IOC borders (arrowheads) but reduced at the PPC-IOC border (arrow). Rst was undetectable at wild type IOC-IOC borders (open arrowheads). Single IOCs (asterisks) and bristle groups (carets) are indicated. B–B″′) *N^ICD^* (B′) was over-expressed in a single IOC and the *rst* transcript (B) was assessed using a *rst* reporter (*rst^F6^-lacZ*). Merged view is shown in B″ and cell shape was visualized using an anti-DE-cadherin antibody (B″′). Arrows point to IOCs that lost lacZ staining. C–C″) Notch is required to suppress *rst* transcription. In the *N^fa-g^* mutant, the *rst* transcript was detected in cells adjacent to cone cells (open arrowheads). Cell morphology was visualized using an anti-Armadillo (Arm) antibody (C). *rst* transcription was assessed using the *rst* reporter *rst^F6^-lacZ* (C′). The merged view is shown in C″. D–D″) *N^ICD^* (green, D′) was over-expressed in a single IOC and the eye was stained with an anti-Kirre antibody (D). The merged view is shown in D′. The enlarged view of a boxed region in D′ is shown in D″. For clarity, only Kirre channel is shown in D″. The Kirre protein was increased at an IOC-IOC border (arrowhead) but reduced at the PPC-IOC border (arrow). Kirre was undetectable at wild type IOC-IOC borders (open arrowheads). Single IOCs (asterisks) and bristle groups (carets) are indicated. Scale bars, 10 µm.

When Notch was activated in a single IOC by expressing *N^ICD^*, as expected, the Rst level was increased at IOC-IOC borders (Fig. 4A–A″′). Unexpectedly, the Rst level was reduced 40% at IOC-PPC borders (Fig. 4A″′ and Table 1). To test whether a reduction of the Rst protein seen at the PPC-IOC border is due to a reduction in *rst* transcription, a *rst* reporter (*rst^F6^-lacZ*) was used to monitor the *rst* activity. Upon activation of Notch in a single IOC, *rst^F6^-lacZ* was lost in the target cell (Fig. 4B–B″′), indicating Notch is sufficient to suppress *rst* transcription in IOCs. Since Notch is normally activated in PPC precursors, this result suggests that in the wild-type eye Notch suppresses *rst* in developing PPCs. Consistently, in *N^fa-g^* mutants, *rst^F6^-lacZ* was expanded to all pigment cells surrounding cone cells (Fig. 4C–C″), indicating that Notch is necessary for suppressing *rst* in emerging PPCs. A similar effect was also seen with *kirre* when Notch activities were altered (Fig. 4D–D″ and Table 1). Taken together, these results indicate that Notch is both sufficient and necessary to suppress *rst* and *kirre* transcription in developing PPCs.

### 5. Distribution dynamics of adhesion molecules

**Figure 5:**
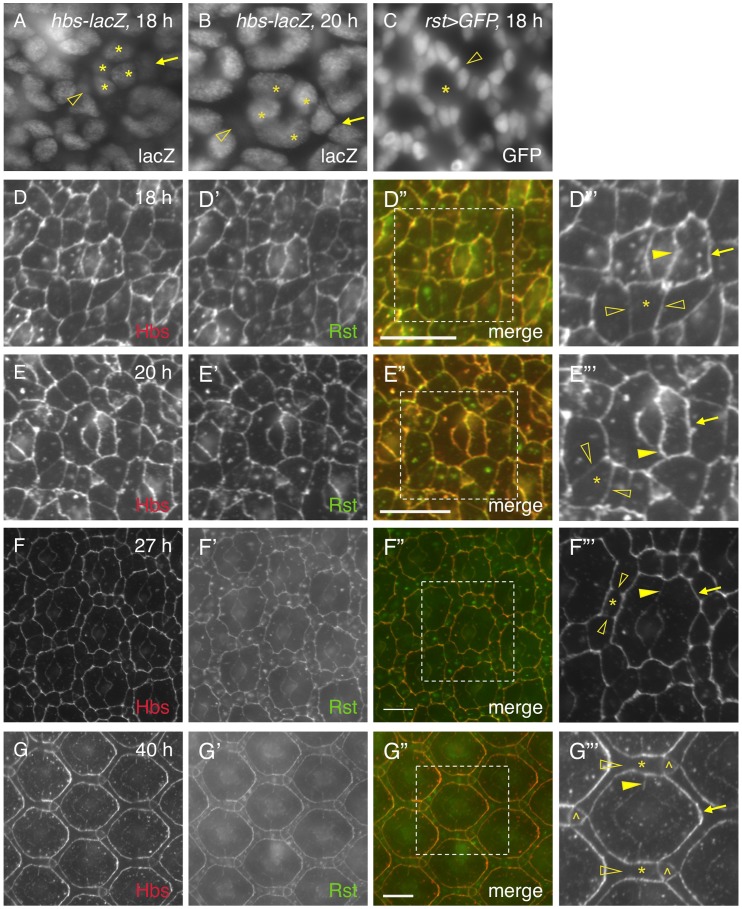
Distribution of Hbs and Rst is dynamic in the eye epithelium. The activity of a *hbs* reporter was assessed using an anti-lacZ antibody (A–B). Cone cells are marked (asterisks). A) The activity of the *hbs* reporter was detected in emerging PPCs at 18 h APF. The nucleus of a PPC precursor (arrow) was rising half way to the level of cone cell nuclei while the nucleus of the second PPC precursor was lagging behind (open arrowhead). B) The activity of the *hbs* reporter was detected in PPC precursors at 20 h APF. The nucleus of a PPC precursor (arrow) had arisen to the level of those of cone cells while the nucleus of the second PPC precursor was still below the plane (open arrowhead). C) *rst* was transcribed in IOCs (open arrowhead) at 18 h APF. *rst-Gal4* was used to drive expression of nuclear GFP(*rst>GFP*). The location for an ommatidium is indicated (asterisk). D–G″′) Distribution of Hbs and Rst is dynamic during 18–40 h APF. The Hbs channels (red) are shown in D–G and the Rst channels (green) in D′–G′. The merged views are shown on D″–G″. The enlarged views of boxed regions in D″–G″ are shown in D″′–G″′. For clarity, only Hbs channels are shown in D″′–G″′. Single IOCs (asterisks) and bristle groups (carets) are indicated. D) At 18 h APF, both Hbs and Rst were found at all borders surrounding PPC precursors including PPC-IOC borders (arrows) and PPC-cone borders (arrowheads). These proteins were also present at IOC-IOC borders (open arrowheads). E) At 20 h APF, PPCs fully enwrapped cone cells. Both Hbs and Rst were enriched at PPC-IOC (arrows), PPC-cone and PPC-PPC borders (arrowheads) while these proteins were slightly reduced at IOC-IOC borders (open arrowheads). F) At 27 h APF, both Hbs and Rst were enriched at PPC-IOC borders (arrows) while these proteins were reduced at PPC-PPC and PPC-cone borders (arrowheads). Hbs and Rst were diminished at IOC-IOC borders (open arrowheads). G) At 40 h APF, both Hbs and Rst proteins were enriched at IOC-PPC borders (arrows) while they were diminished at PPC-PPC and PPC-cone borders (arrowhead). Hbs and Rst were undetectable at IOC-IOC borders (open arrowheads). Scale bars, 10 µm.

It has been shown previously that genes coding for adhesion molecules of the IRM family are expressed in complementary cell types during cell rearrangement (e.g., 24 h APF): *hbs* and *sns* in PPCs; *rst* and *kirre* in IOCs [14], [23]. The patterns of *hbs* and *rst* transcription at 18 h are similar to those at later stages (e.g., 27 h APF) based on the *hbs* and *rst* reporters (Fig. 5A–C). However, immune-staining using specific antibodies revealed striking differences in the distribution patterns of the Hbs and Rst proteins in the eye between 18 h and 40 h APF. Especially, both Hbs and Rst were present ubiquitously at a high level at all borders among epithelial cells at 18 h APF (Fig. 5D–D″′). This is in drastic contrast to later stages (e.g., 27–40 h APF) when both Hbs and Rst were diminished at IOC-IOC and PPC-cone borders (see below). At 20 h APF when two PPCs fully enwrapped the four cone cells, both Hbs and Rst remained at a high level at PPC-PPC and PPC-cone borders but slightly reduced at IOC-IOC borders (Fig. 5E–E″′). At 27 h APF, similar to earlier stages, both Hbs and Rst were enriched at PPC-IOC borders. In contrast, these proteins were reduced at PPC-PPC and PPC-cone borders and diminished at IOC-IOC borders (Fig. 5F–F″′). At 40 h APF, both Hbs and Rst proteins were again enriched at IOC-PPC borders but diminished at PPC-PPC and PPC-cone borders (Fig. 5G–G″′). They were undetectable at IOC-IOC borders (Fig. 5G–G″′). A similar dynamics in protein distribution was also observed with Sns and Kirre (data not shown). These results indicate that four adhesion molecules are initially present in all epithelial cells at 18–20 h APF in the eye and removed from one group of cells at later stages. Therefore, distribution of Hbs, Sns, Rst and Kirre proteins undergoes a transition from ubiquitous to complementary distribution during epithelial remodeling.

### 6. *cis*-interactions destabilize the adhesion complex

**Figure 6:**
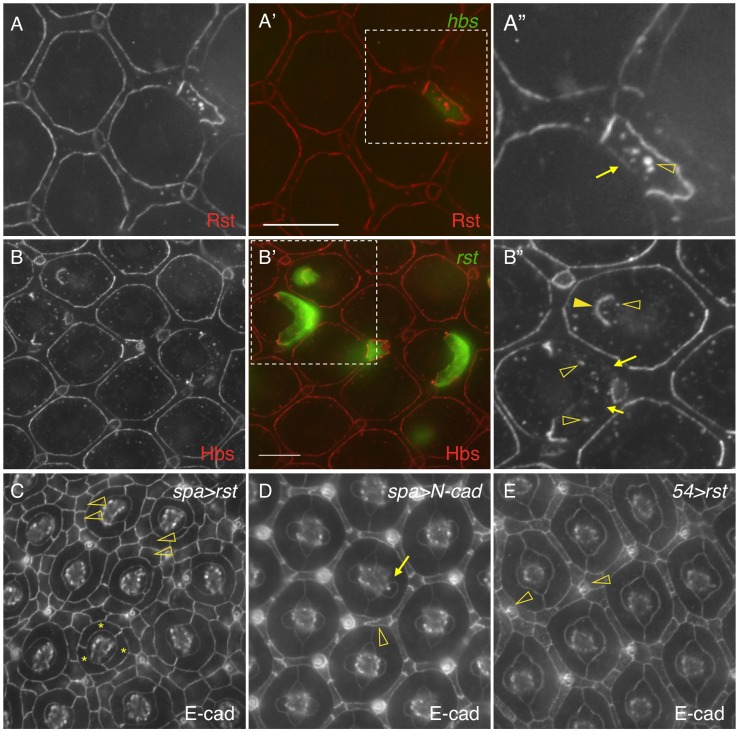
*cis*-interactions promote protein turnover. A–A″) Hbs promotes turnover of Rst in the same cell. *hbs* was mis-expressed in a single IOC (A′) and the eye was stained with an anti-Rst antibody (A). Levels of Rst were reduced at the border between the target IOC and its neighboring PPC (arrow) and increased in vesicles (open arrowhead). Merged view is shown in A′. The enlarged view of a boxed region in A′ with the Rst channel is shown in A″. B–B″) Rst promotes turnover of Hbs in the same cell. *rst* was mis-expressed in single cells (B′) and the eye stained with an anti-Hbs antibody (B). The Hbs level was reduced at PPC-IOC borders (arrows). A higher level of the Hbs protein was observed in vesicles (open arrowheads). When Rst was expressed in a cone cell, the Hbs level was elevated at the cone-PPC border (arrows). The target cone cell also had a higher level of Hbs in vesicles (open arrowheads). Merged view is shown in B′. The enlarged view of a boxed region in B′ with the Hbs channel is shown in B″. C) Interference of Hbs by mis-expressing Rst in PPCs (*spa>rst*) led to severe disruption of the hexagonal pattern of the eye. Three cells surrounding a cone cell cluster are highlighted (asterisks). IOCs failed to sort into a single file (open arrowheads). D) Over-expression of N-cadherin in cone cells (*spa>N-cadherin*) had a mild effect on tissue remodeling. An abnormal cone cell was highlighted (arrow) along with a defective IOC (open arrowhead). E) Over-expression of Rst in IOCs (*54>rst*) had a mild effect on tissue remodeling. Several IOCs formed a cluster around a bristle group (open arrowheads). Scale bars, 10 µm.

Hbs and Sns from the Nephrin group and Rst and Kirre from the Neph1 group co-localize at the border between PPCs and IOCs, and heterophilic interactions between these two groups of proteins *in trans* (interactions between proteins from two adjacent cells or *trans*-interactions) stabilize the adhesion complex on the membrane [14], [23]. The observation that both Hbs and Rst were found in all IOCs at the beginning of cell rearrangement (Fig. 5D–E″′) raises the question of how these IRM adhesion molecules interact with each other when placed in the same cell (*cis*-interaction). To assess the effect of *cis*-interaction, Hbs was mis-expressed in the cells that normally express the counterparts Rst and Kirre. Upon expression of Hbs in a single IOC, the level of Rst was reduced about 63% at the PPC-IOC border and the number of vesicles was increased significantly in the target IOC (Fig. 6A–A″ and Table 1). Nevertheless, transcription of *rst* as assessed by the *rst* reporter *rst^F6^-lacZ* was not altered in the clone (data not shown). Similarly, when Rst was mis-expressed in a single PPC that normally transcribes *hbs* and *sns*, the Hbs level on the membrane was reduced 93% and the number of vesicles increased markedly in the target PPC (Fig. 6B–B″ and Table 1). Similarly, the activity of the *hbs* reporter *P[w+]36.1* was unchanged in the clone (data not shown). These results suggest that, while heterophilic interactions between two groups of IRM adhesion molecules *in trans* stabilize both proteins on the membrane, interactions among these proteins *in cis* destabilize proteins on the membrane and promote turnover of these proteins.

To assess the effect of *cis*-interactions on pattern formation, *rst* was mis-expressed in all PPCs using *spa-Gal4*. Spa-Gal4 is known to drive expression of transgenes in cone cells and PPCs [25]. Upon expression of *rst* in cone cells and PPCs (*spa>rst*), the hexagonal pattern of the eye was severely disrupted. While spatial organization of cone cells was mildly affected, various numbers of PPCs (typically ranging from 1 to 3) were found adjacent to cone cells. More strikingly, IOCs failed to sort into single file. As a result, 2–3 rows of IOCs scattered in between ommatidia across the eye and the eye was extremely rough (Fig. 6C). To exclude the possibility that the effect of over-expression was simply due to enhanced adhesion among cone cells and/or PPCs, N-cadherin was over-expressed in these cells using the same *spa-Gal4*. N-cadherin is known to mediate adhesion among cone cells through homophilic interactions [12]. In contrast to Rst, over-expression of N-cadherin (*spa>N-cadherin*) only led to mild defects in IOCs and cone cells with largely intact PPCs (Fig. 6D). To exclude the possibility that the severe defects seen in *spa>rst* are simply due to detrimental effects of the protein on the cells when expressed at a high level, Rst was over-expressed in IOCs using *Gal4-54* (*54>rst*). In the *54>rst* eye, IOCs were occasionally found in cluster with a bristle group. Nevertheless, the hexagonal pattern was only mildly affected (Fig. 6E). Although we cannot exclude the possibility that different protein levels also contribute to the different phenotypes seen in these experiments, the results presented here strongly suggest that interference of IRM adhesion molecules by *cis*-interactions has a strong impact on the establishment of the hexagonal pattern.

### 7. Adhesion restores spatial pattern of cell clusters

**Figure 7:**
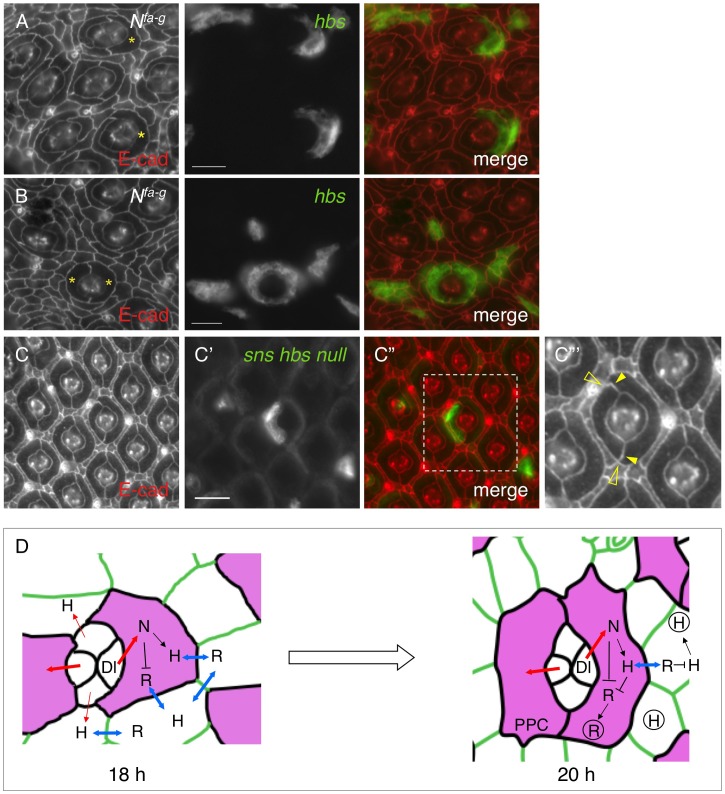
Spatial organization of primary pigment cells requires Hbs and Sns. Eyes were stained with an anti-E-cadherin antibody (red, left). Target (mutant or over-expression) cells are marked by GFP (green, middle). The merged views are shown on the right. A–B) Expression of Hbs in a single cell restored the ‘kidney’ shape of PPCs. In the *N^fa-g^* eye, *hbs* was expressed in single IOCs. When *hbs* was expressed in single cells adjacent to cone cells, the cell (asterisks) spread around cone cells (A). When *hbs* was introduced into two cells adjacent to cone cells, these cells (asterisks) fully enwrapped cone cells (B). C–C″′) Hbs and Sns are required for organization of PPCs. In a single PPC mutant for both *hbs* and *sns* (green, C′), the cell reduced the apical surface area and PPC-IOC border. The enlarged view of a boxed region in C″ with the E-cadherin channel is shown in C″′. Open arrowheads mark the shortened PPC-IOC border while arrowheads highlight the curved PPC-PPC borders. D) A model for control of PPC recruitment by cell signaling and cell adhesion. At 18 h APF, all IOCs that contact cone cells have access to Dl and express Hbs. However, IOCs adjacent to anterior-posterior cone cells receive a high level of Notch signaling (thick red lines) than other IOCs (thin red lines). These cells express a higher level of Hbs than other IOCs. Hbs boosts the ability of these cells to enwrap cone cells and gain more access to Dl. Therefore, Notch signaling and Hbs create a positive feedback loop so that initially a small difference in Notch signaling is amplified. As a result, two cells adjacent to anterior and posterior cone cells outcompete other IOCs and enwrap cone cells as PPC precursors. At 20 h APF, PPC precursors gain full access to Dl and constantly produce Hbs while shutting down Rst production. The remaining Rst in PPCs is removed by *cis*-interactions. In the meantime, other IOCs that are now denied access to Dl constantly supply Rst. The remaining Hbs in IOCs are cleared out by *cis*-interactions, leading to complementary distribution of two groups of adhesion molecules. For simplicity, only Hbs and Rst are shown. H = Hbs; R = Rst; N = Notch; Dl = Delta. Double-headed arrows represent *trans*-interactions between Hbs and Rst; Hbs and Rst in circle represent proteins in vesicles. Scale bars, 10 µm.

This work demonstrates that Notch controls transcription of IRM adhesion genes. On the other hand, Notch is also known to control transcription of multiple other genes during eye development. It is not clear whether loss of adhesion molecules is responsible for the PPC defects seen in Notch mutants. To address this issue, a rescue experiment was performed using *N^fa-g^* as a background mutant and *UAS-hbs* as a rescue construct. When Hbs was expressed in a single cell adjacent to cone cells, 83% of the target cells (n = 257, 13 eyes) elongated and the interface between the target cell and IOCs expanded in a manner similar to a wild type PPC (Fig. 7A). Further, when Hbs was expressed in two cells adjacent to cone cells, in nearly all cases examined so far, Hbs positive cells fully enwrapped the cone cell group from the anterior and posterior sides resembling two wild type PPCs (Fig. 7B). These results indicate that adhesion is sufficient to restore spatial relationship of cell clusters in Notch mutants.

To test the necessity of cell-cell adhesion for formation of the spatial pattern of PPCs, *hbs* and *sns* double mutant was generated using *sns^ZF1.4^* and *hbs^459^* mutant alleles (see Materials and Methods). Large clonal patches generated using this double mutant together with *ey-FLP* led to extremely rough eyes in adults (data not shown). Single PPCs mutant for both *sns* and *hbs* had a shorten PPC-IOC border and reduced apical surface (Fig. 7C–C″′). In addition, PPC-PPC border became curved. As a result, the apical profile of the target PPC became more rounded. These results indicate that *sns* and *hbs* are required for the normal ‘kidney’ shape of PPCs.

## Discussion

In developing tissues, one way to isolate a small group of cells from other groups is to induce a few neighboring cells to surround them. This paper shows that Notch provides an instructive signal in inducing neighboring cells to spread around and eventually surround centrally localized cone cells in the *Drosophila* eye. This work demonstrates that Notch functions in this process by differentially regulating four adhesion genes.

### Notch controls cell adhesion

This work demonstrates that Notch is involved in cell-cell adhesion by regulating transcription of adhesion genes. Notch signaling is known to play a pleiotropic role in controlling cell fate during animal development [26]. The requirement of Notch during *Drosophila* embryonic development after cell fate decision has led to speculation that Notch is involved in cell adhesion [5]. This notion is supported by the behavior of PPCs in the pupal eye [4]. However, clear evidence linking Notch to cell adhesion has been lacking. This study shows that in the pupal eye Notch differentially controls transcription of four IRM adhesion genes. Notch activates transcription of *hbs* and *sns* but represses *rst* and *kirre*, leading to differential expression of IRM adhesion genes in two populations of cells: IOCs by default express *rst* and *kirre*; PPCs by activation of Notch signaling express *hbs* and *sns*. Heterophilic interactions between Hbs/Sns and Rst/Kirre proteins mediate preferential adhesion between IOCs and PPCs [14], [23]. Therefore, Notch signaling sets up differential expression of adhesion genes (Fig. 7D).

This work also illustrates how a single signaling pathway transforms an initially homogeneous population of cells into two morphologically distinct groups of cells. In the wild-type eye, PPCs are polarized since PPCs without exception enwrap cone cells from anterior/posterior rather than from polar/equatorial sides. Data presented in this work suggest that asymmetric distribution of Dl in cone cells sets up PPC polarity. At the beginning of cell rearrangement (∼18 h APF), all IOCs that contact cone cells have access to Dl and express Hbs. However, asymmetric expression of Dl in cone cells creates a bias. IOCs that contact anterior-posterior cone cells receive a high level of Notch signaling (thick red lines, Fig. 7D) and produce more Hbs, which in turn boosts the ability of these cells to enwrap cone cells and gain more access to Notch signaling. In contrast, other IOCs that initially receive a low level of Notch signaling (thin red lines, Fig. 7D) are at a disadvantage and quickly lose competition to PPC precursors in enwrapping cone cells. As a result, Notch and Hbs create a positive feedback loop through which an initial small difference in Notch signaling is amplified, giving rise to PPCs exclusively enwrapping cone cells from anterior and posterior sides (Fig. 7D).

### *cis*-interactions promote protein turnover

This work provides evidence that interactions between adhesion molecules from the Nephrin group and those from the Neph1 group *in cis* promote protein turnover. IRM adhesion molecules are known to form heterophilic interactions. Proteins from the Nephrin group bind *in trans* to proteins from the Neph1 group and *trans*-interactions among IRM adhesion molecules stabilize proteins on the membrane [14], [23]. In contrast, *cis*-interactions among these proteins destabilize proteins on the membrane (this work). Results presented herein support a model that *cis*-interactions provide a mechanism for removing counterpart proteins from the same cells (Fig. 7D). After two PPC precursors completely surround the cone cell group (e.g., 20 h APF), these cells gain full access to Dl. In response to Notch signaling, PPC precursors constantly produce Hbs, which removes Rst from the same cells through *cis*-interaction. By the same mechanism, all other IOCs that are now denied access to Dl by default constantly produce Rst, which in turn clears Hbs from IOCs. Therefore, a combination of transcriptional regulation by Notch and post-translational mechanism by *cis*-interactions provides a mechanism for the transformation of initially ubiquitous distribution into complementary distribution of four adhesion molecules within two populations of cells (Fig. 7D).

*cis*-interactions observed in the *Drosophila* eye are very reminiscent of interactions between the Notch receptor and its ligand Dl. It has been shown that, in the *Drosophila* embryo and the eye imaginal disk, an increase of Dl in a Notch-expressing cell inhibits Notch signaling in a cell-autonomous fashion via *cis*-interaction [27], [28]. In the Notch-Dl case, the level of Dl within a Notch-expressing cell determines the intensity of Notch signaling that cells receive, which in turn determines cell fates [27], [28]. In the case of IRM adhesion molecules, the level of a protein from one group in a cell determines the amount of counterpart proteins from the other group on the membrane of the same cell, which alters cell-cell adhesion. More specifically, in the *Drosophila* eye *cis*-interactions remove remnant proteins and facilitate the differential distribution of IRM adhesion molecules without affecting cell fate. Despite different impact of *cis*-interactions on cell-cell interactions in both cases (N-Dl versus IRM adhesion molecules), they share one common feature: presence of one protein interferes with the function of the counterpart protein in the same cell. What structural elements are involved in *cis*-interactions between these proteins and how *cis*-interactions lead to a reduction of protein activity still remain questions for further investigation.

Although evidence presented in this work suggests a simple relationship among cell signaling, cell adhesion and cell shape, two observations highlight the complexity of PPC recruitment in the developing *Drosophila* eye. First, *hbs* can restore the ‘kidney’ shape of PPCs at a lower frequency than *Notch* (or *N^ICD^*) in the *N^fa-g^* mutant. This observation suggests that adhesion genes may not represent all the function of *Notch* in recruiting PPCs. Notch is known to have a wide range of target genes. In particular, several transcription factors are known targets of Notch for the determination of PPC cell fate [29]. Therefore, it is possible that additional effectors of *Notch* signaling are also involved in conferring on PPCs the ability to enwrap cone cells. Second, this work suggests a positive feedback loop that promotes selection of PPC precursors in the developing eye (Fig. 7D). On the other hand, it has been shown recently that Hbs promotes Notch signaling by interacting with presenilin [30]. A potential more direct impact of Hbs on Notch signaling raises the possibility that there may exist a second positive feedback loop between Notch and Hbs: Notch activates *hbs* transcription and Hbs in return enhances Notch signaling, whereby initially a small difference of Notch signaling among IOCs is amplified, leading to separation of Hbs/Sns-expressing cells from those expressing Rst/Kirre. Whether the second positive feedback loop plays a role in PPC recruitment remains to be tested.

This paper illustrates how a small number of cells utilize a single signaling pathway to instruct neighboring cells to surround them, whereby the centrally localized cells are isolated from other cells. Since isolation of a group of cells by another is commonly seen in developing tissues, a correlation between cell signaling and cell adhesion may be a more general mechanism for organizing cells during organ formation.

## Materials and Methods

### 1. *Drosophila* genetics

The *sns* and *hbs* double mutant *sns^ZF1.4^ hbs^459^* was generated for this work by recombining *sns^ZF1.4^* and *hbs^459^*, a loss-of-function allele of *sns* and *hbs*, respectively, onto the second chromosome. *N^fa-g^*, *UAS-Notch RNAi*, *Dl-lacZ*, *y w hsFLP*, *UAS-nlsGFP* and *Act5C>y+>Gal4 UAS-GFP* were provided by the Bloomington Stock Center. *rst-Gal4* was obtained from National Institute of Genetics Fly Stock Center (Japan). Other flies used: *rst^F6^-lacZ* [31], *spa-Gal4* [25], *UAS-N-cadherin* [12], *sns^ZF1.4^* and *UAS-sns* (gift of Susan Abmayr), *UAS-N^ICD^* (gift of Cedric Wesley), *UAS-N^ICD^-lexA* (gift of Toby Lieber), *P[w+]36.1* and *hbs^459^* (gift of Mary Baylies), *UAS-hbs* (gift of Helen Sink), *GBE-Su(H)_m8_-lacZ* (*N-lacZ*) [22], *Gal-54* [23], *UAS-rst* (gift of Karl-F. Fischbach), *UAS-kirre/duf* (gift of Marc Ruiz-Gomez), *UAS-Dl* (gift of Marek Mlodzik) and *hsFLP MKRS* (gift of Matthew Freeman).

### 2. Clonal analyses

Single cell clones for over-expressing a target gene were generated using a FLP-out technique as described previously [14]. To induce clones, pupae at 12 h APF were heat-shocked at 37°C in a water bath for 20 min. Clones were marked by GFP. The genotypes of clones are shown as follows:

1. *UAS-N^ICD^/Act5C>y+>Gal4 UAS-GFP; hsFLP MKRS/+* (Figs. 3A, 4A and 4D)
2. *P[w+]36.1/Act5C>y+>Gal4 UAS-GFP; UAS- N^ICD^/hsFLP MKRS* (Fig. 3B)
3. *UAS-Dl/Act5C>y+>Gal4 UAS-GFP; hsFLP MKRS/+* (Figs. 3C–D)
4. *N^fa-g^; P[w+]36.1/Act5C>y+>Gal4 UAS-GFP; UAS- N^ICD^/hsFLP MKRS* (Fig. 3E)
5. *N^fa-g^; UAS- N^ICD^/Act5C>y+>Gal4 UAS-GFP; hsFLP MKRS/+* (Fig. 3F)
6. *rst^F6^-lacZ/Act5C>y+>Gal4 UAS-GFP; UAS- N^ICD^/hsFLP MKRS* (Fig. 4B)
7. *UAS-hbs/Act5C>y+>Gal4 UAS-GFP; hsFLP MKRS/+* (Figs. 6A)
8. *UAS-rst/Act5C>y+>Gal4 UAS-GFP; hsFLP MKRS/+* (Figs. 6B)
9. *N^fa-g^; UAS-hbs/Act5C>y+>Gal4 UAS-GFP; hsFLP MKRS/+* (Fig. 7A–B)

Loss-of-function clones were generated using a MARCM technique [32]. Clones were induced by heat-shocking third instar larvae at 37°C for 1 h. Clones were marked by GFP. The genotype of clones: *yw hsFLP; FRT42D sns^ZF1.4^ hbs^459^/FRT42D Gal80; tub-Gal4 UAS-mCD8-GFP/+* (Fig. 7C).

### 3. Immunohistochemistry

Immunostaining of the pupal eye was carried out as described [14]. Rat anti-Kirre (1∶5000) and rabbit anti-Hbs AS14 (1∶2500) were used as previously described [23]. Other primary antibodies: mouse anti-Rst Mab24A5.1(1∶100) [33], rabbit anti-Sns (1∶300) [34] and rabbit anti-lacZ (1∶2000; 5 Prime→3 Prime). Rat anti-DE-cadherin (1∶20), mouse anti-Armadillo (1∶20) and mouse anti-Dl 9B (1∶20) were provided by Developmental Studies Hybridoma Bank at the University of Iowa. Secondary antibodies: Alexa 488 and Alexa 568 conjugated secondary antibodies (1∶5000; Molecular Probes); Cy5 conjugated secondary antibodies (1∶1000; Jackson ImmunoResearch Laboratories). All images were captured using an Axioplan2 epi-fluorescence microscope equipped with an Axiocam digital camera (Carl Zeiss, Inc.).

### 4. Quantification of membrane protein levels

Levels of membrane proteins were quantified using ImageJ [35]. Briefly, a long and narrow stripe that surrounded and closely followed the target border was carefully traced. The integrated density (ID_1_) of the selected region was recorded using ImageJ. The background integrated density (ID_0_) was recorded by moving the same selection box to a background region. The membrane protein level (I) reflected by integrated intensity per unit length was determined following I = 2*(ID_1_−ID_0_)/L, where L is the perimeter of the selected region in pixel. For each experiment, the average intensity from 6 neighboring wild type borders was calculated and used as a control.
